# Global Cultural Change and Anxiety in Children and Adolescents: Analyzing Socialization Goals Over Three Decades in 70 Countries

**DOI:** 10.1111/desc.70157

**Published:** 2026-03-08

**Authors:** Leonard Konstantin Kulisch, Ana Lorena Domínguez Rojas, Silvia Schneider, Babett Voigt

**Affiliations:** ^1^ Mental Health Research and Treatment Center (FBZ) Ruhr University Bochum Bochum Germany; ^2^ German Center for Mental Health (DZPG) Bochum/Marburg Germany; ^3^ Philosophy of Mind and Cognition Osnabrück University Osnabrück Germany; ^4^ Catholic University of Pereira Pereira Colombia

**Keywords:** individualism/collectivism, longitudinal analysis, national culture, prevalence, psychiatric disorders, public health

## Abstract

On average, cultures seem to shift towards a greater emphasis of an independent social orientation. However, this shift may vary, with some cultures following different trajectories. Cultural transformations also affect the norms regarding the qualities favored in children, known as socialization goals. Research suggests that a greater shift towards independence may have more negative consequences for child and adolescent mental health. This study explored links between time period changes (1989–2022) in socialization goal norms and the child and adolescent anxiety disorder incidence rates across 70 countries. The analysis drew on data from the World Value Survey, Global Burden of Disease study, Human Development Report, and Future of Families and Child Wellbeing Study. Country‐level cross‐temporal regression revealed no global effect of independence‐oriented socialization norms. Moderation analyses showed no association among non‐WEIRD (Western, Educated, Industrialized, Rich, Democratic) countries but a significantly stronger link between independence orientation and more anxiety disorders among WEIRD countries. Globally, more emphasis on the interdependence‐related goal “religious faith” was associated with fewer anxiety disorders across time. Validation analyses with individual‐level data from the United States replicated this finding and showed that religiosity norms were a stronger predictor than maternal religiosity. A cross‐lagged panel model confirmed potential effects from religiosity to child and adolescent anxiety. Although effect sizes were small, these findings suggest that religiosity may serve as a protective factor by fostering a sense of purpose and social connectedness, globally. This study highlights the potential role of cultural change in children's and adolescent's mental health and underscores the need for greater support for young people growing up in increasingly secular societies.

**Summary:**

- This study tested links between cultural changes in socialization goals and anxiety disorder incidence rates among children and adolescents over three decades.
- Independence‐related socialization goal norms were not globally linked to anxiety disorders, but their adverse effect appeared specific to WEIRD countries.
- The importance of religious faith in socialization, linked to interdependence, was associated with fewer anxiety disorders across 70 countries.
- Religiosity norms were also related to child and adolescent anxiety symptoms on individual level and showed expected directional effects.

## 1 Introduction

In the 1980s, parenting guidebooks by James C. Dobson like “Dare to Discipline” advocating strict parenting that aimed to foster obedience and compliance sold millions of copies. Nowadays, such books are mostly banished to the back corners of thrift stores. Current top sellers include “Parenting with Love and Logic: Teaching Children Responsibility” by Foster Cline and Jim Fay. Their book places importance on very different values that children and adolescents should develop compared to “Dare to Discipline.” These books illustrate how culture and socialization have changed over the past decades. Societies around the world seem to develop towards independent models of socialization (e.g., encouraging feelings of responsibility) while shifting away from interdependent models of socialization (e.g., encouraging obedience). This raises the question how such societal transformations impact the lives of children and adolescents. This study aims to shed light on the interplay between culture and mental health of children and adolescents by delving into the relationship between changes in socialization goal norms, particularly those aligned with an independent or interdependent social orientation, and the incidence of child and adolescent anxiety disorders across countries.

### 1.1 Culture and Socialization

Culture provides guiding principles through collectively upheld norms that help make sense of the complex environment that children are born into (Cialdini et al. 1991). Culture has a polysemic character (Kroeber and Kluckhohn 1952). This research explores the symbolic component of culture that refers to a way to interpret the world (Keller 2007) by providing “goals, values, and pictures of the world or ideas about what is true, good, beautiful, and efficient” (p. 11; Shweder 2003).

Cultural norms and social values are passed down through generations in the process of socialization. Adults adopt social values as desirable and strive to cultivate them in the children growing up in a respective culture (Keller and Lamm 2005; Lavelli et al. 2019). Concretely, the socialization process occurs through child‐rearing and educational practices (Rossano 2012). *Socialization goals* are the answer to the question ‘What outcomes do guidebook authors, politicians, teachers, priests, and parents hope to achieve in raising and educating children?’ Such shared goals are the aspect of culture that is explicitly directed towards children and adolescents.

Socialization goals materialize in structural factors of the environment, such as parenting guidebooks, corporal punishment laws, school curricula, or religious practices like infant baptism (Coninx and Stephan 2021). They are also expressed in specific behaviors of parents, grandparents, or teachers aimed at fostering certain qualities of character or traits in children (Keller and Kärtner 2013). Developmental science has provided strong evidence for the crucial role of socialization goals for human development. They mold early affectivity, relational styles, and self‐esteem (Buhler‐Wassmann and Hibel 2021; Hewlett and Lamb 2002; Tronick and Beeghly 2011). Studies have shown that socialization goals can potentially influence the cognitive (He et al. 2021), social (Kärtner et al. 2022), and emotional development of children and adolescents (Lavelli et al. 2019). This has potential implications for mental health, and, therefore, socialization goals deserve special attention in developmental research.

### 1.2 Cultural Dimensions

Different scholars have tried to map global cultural differences using broad cultural dimensions. Varnum et al. (2010) suggested to summarize findings from different domains of cognition, emotion, and behavior into a unified framework. The authors propose one cultural dimension: independent social orientation vs. interdependent social orientation. Each culture but also each individual can be located somewhere between these two poles (Tamis‐LeMonda et al. 2008). This integrates various cross‐cultural theories. Independent self‐construals (Markus and Kitayama 1991), individualistic values (Triandis 1996), and socially disengaging emotions (Kitayama et al. 2006) are aspects of an independent social orientation. On the other end, interdependent self‐construals (Markus and Kitayama 1991), collectivistic values (Triandis 1996), and socially engaging emotions (Kitayama et al. 2006) are aspects of an overall interdependent social orientation. Cultures with a more independent social orientation have been located especially in high‐income European and English‐speaking countries which are referred to as *WEIRD* (i.e., Western, educated, industrialized, rich, and democratic) countries (Henrich et al. 2010). Cultures with a more interdependent social orientation have been typically associated with Asian, African and Arab countries (Parkinson et al. 2005).

A data‐driven analysis of the socialization goal norms across 55 countries in the fifth wave of the World Value Survey (Inglehart et al. 2022) showed the same country‐level differences (Bond and Lun 2014). A main dimension of socialization goal variation explained about 36% in global differences: One end of this dimension was marked by independence, feeling of responsibility, and imagination—qualities linked to an independent social orientation. The other end was characterized by religious faith, obedience, and hard work—qualities linked to an interdependent social orientation. This empirical finding is consistent with the framework of Varnum et al. (2010). Global socialization goal norm variation can be described on a broad dimension ranging from an emphasis on independence to an emphasis on interdependence. Independence socialization goals were more commonly endorsed in WEIRD countries, whereas interdependence socialization goals were more frequently endorsed in non‐WEIRD countries (Bond and Lun 2014).

It is important to differentiate independence and interdependence from the human needs for autonomy (i.e., willingly endorsed behavior) and relatedness (i.e., belonging, attachment, intimacy) often discussed in cultural research (Kagitcibasi 2005). Basic needs can be satisfied in both independent and interdependent cultures (Chirkov et al. 2003). While broad cultural dimensions enable a meaningful picture of cultural variance, they may oversimplify global variation. For example, a large study found that some context‐specific independent and interdependent self‐construals can co‐exist within the same culture (Vignoles et al. 2016). This highlights the importance of empirically testing which dimensions capture variation in each dataset (such as by conducting factor or principal component analyses) rather than assuming that predefined cultural dimensions would always apply. Moreover, the role of the complex dynamics within social contexts should not be neglected. Despite the importance of the distinction between independence and interdependence for understanding the socialization process, it is important to avoid over‐simplifying the perspective and consider that rearing may be influenced by other cultural norms (Wong et al. 2020).

### 1.3 Cultural Change

It is important to note that cultural differences are not cast in stone but that culture is rather a dynamically changing system. Empirical evidence from a range of cognitive, emotional and behavioral domains suggests an average cultural shift towards a more independent social orientation and away from an interdependent one, globally (Cai et al. 2019). A country‐level analysis by Santos et al. (2017) showed that between 1960 and 2011, 39 out of 53 countries underwent a transition towards greater endorsement of values associated with an independent social orientation (e.g., preference for self‐expression).

For socialization goals, a corresponding pattern was found among parents within the World Value Survey (Inglehart et al. 2022). Cross‐sectionally, generational differences pointed towards a shift towards a greater importance of independence‐related socialization goals. Compared to older generations, younger parents placed a stronger emphasis on independence. At the same time, interdependence became less important. This was evident across WEIRD and East Asian countries (Park et al. 2014).

However, more recent literature suggests that this may be an oversimplified view on cultural change. An analysis using similar data by Jackson and Medvedev (2024) revealed that not all cultures show the same magnitude of shift towards an independent social orientation. In fact, some cultures even seemed to be transitioning away from such values.

### 1.4 Culture and Mental Health of Children and Adolescents

Cultural change towards a more independent social orientation goes along with negative (population‐level) physical and mental health consequences according to the cultural fraud hypothesis (Eckersley 2006). Eckersley (2006) defined cultural fraud as “the promotion of images and ideals of ‘the good life’ that serve the economy but do not meet psychological needs or reflect social realities” (p. 256). He argued that modern Western culture characterized by a strong independent social orientation was harmful. Such norms about independence and self‐orientation may have been beneficial for the economic growth and respective increase in life quality decades ago by creating innovation, competition, and entrepreneurship. However, this was no longer the case. Eckersley acknowledged the general notion that culture does not develop or change out of nowhere but adapts to features of the physical, social, and political environment (Levine 2007; Rogoff 2003). Yet, already Whiting and Whiting (1960) agreed that socially shared ideals can weigh heavier than adaptation to the environment in some scenarios. Some cultural practices (e.g., those that embody socialization goals) may persist even if they become objectively maladaptive (for further discussion see Greenfield et al. 2003). The rise of strong independence values may have led to more loneliness (Barreto et al. 2021), and increased expectations towards children and young people (Curran and Hill 2022), more competitiveness (Butler 2021), physical inactivity (Akaliyski et al. 2022), and diminished intrinsic motivation (Twenge et al. 2012).

Evidence for the hypothesis comes from a systematic review that summarized empirical studies testing associations between independence values and wellbeing on individual and country levels. The review focused on young people aged 18–29 years mainly living in WEIRD countries. Out of the 14 reviewed studies, 10 found at least some links between independence values and low wellbeing or interdependence values and high wellbeing. However, all studies used cross‐sectional designs, and no actual cultural change was investigated (Humphrey and Bliuc 2021).

To sum up, socialization goal norms describe socially shared child qualities that members of a culture strive to cultivate in children and adolescents growing up in a respective culture. Socialization goals shape development and may affect child and adolescent mental health. Cultural differences can be described on the independence‐interdependence dimension. Cultural shifts toward emphasizing independence in socialization are suggested to have negative effects on child and adolescent mental health, as proposed by the cultural fraud hypothesis and supported by cross‐sectional evidence from mostly WEIRD countries. Studies considering cross‐temporal associations across multiple decades are missing. No prior research has specifically examined socialization goal norms—the cultural norms that directly aim to shape human development. Research is needed to cover various world regions besides WEIRD countries and include the “Majority World”, the non‐WEIRD countries in which the majority of humans live but which are underrepresented in research. This will also give insights into cultural mismatch dynamics in cultures traditionally characterized by independence (WEIRD) and interdependence (non‐WEIRD; Fulmer et al. 2010)

### 1.5 Present Studies

In two complementary studies, we aimed to explore the cross‐temporal relationship between changes in socialization goal norms and changes in child and adolescent mental health. We focused on socialization goals as a cultural aspect specifically directed at children and adolescents (Keller and Kärtner 2013). Anxiety disorders and anxiety symptoms were selected as appropriate outcome measures of child and adolescent mental health for the following reasons. First, anxiety disorders (e.g., specific phobias, separation anxiety disorder) are among the first emerging (Solmi et al. 2022) and most common (Polanczyk et al. 2015) mental health issues during childhood and adolescence. Furthermore, anxiety disorders are largely influenced by environmental factors (Burmeister et al. 2008; Gregory and Eley 2007) and their prevalence is sensitive to cultural factors (Hofmann and Hinton 2014). Previous works investigating cultural change focused on few selected time points (Jackson and Medvedev 2024; Park et al. 2014), broad groups of countries (Jackson and Medvedev 2024; Santos et al. 2017), and simple linear increases or decreases between across time (Park et al. 2014; Santos et al. 2017). The present research considered country‐specific patterns of change (i.e., distinct fluctuations across multiple time points) in culture and anxiety. This is needed because of the complexity of cultural trajectories (Jackson and Medvedev 2024) and change patterns in anxiety disorders (Javaid et al. 2023).

Study 1 examined cultural change directly by testing whether country‐level shifts in socialization goal norms predicted changes in the incidence of anxiety disorders. Study 2 built on these findings by focusing on individual‐level mechanisms, investigating how religiosity norms and maternal religiosity relate to child anxiety symptoms, thereby providing insight into how cultural change at the macro level becomes consequential in individual lives.

## 2 Study 1

In the first study, we analyzed country‐level data to cover a broad variance of cultures. In addition to the WEIRD perspective that was studied in most previous works (e.g., Humphrey and Bliuc 2021), we included countries from all continents. Following the cultural fraud hypothesis (Eckersley 2006), we expected positive cross‐temporal correlations between changes in the importance of socialization goals linked to an independent social orientation and changes in the anxiety disorder incidence rate. To establish that any correlations are not solely the by‐product of general social and medical improvements, we tested whether the correlations persisted when controlling for the human development index (Greenfield 2009). Considering the focus on WEIRD countries in previous research (Eckersley 2006; Humphrey and Bliuc 2021), we contrasted the cross‐temporal associations between WEIRD and non‐WEIRD countries. That said, our overarching aim was to identify cultural factors that would be relevant for child and adolescent mental health on a global scale. This design captured relevant time period effects of cultural change as well as cohort effect. Age effects were eliminated as data from children and adolescents aged 0–19 years were aggregated. Because age, period, and cohort are linearly dependent (period = cohort + age), these effects can never be fully disentangled (Rohrer 2025).

### 2.1 Methods

We utilized three publicly available data sets to gather country‐level estimates of socialization goal norms, child and adolescent anxiety disorder incidence rate, as well as social and medical development. The data sources were selected based on the available variables, the timeframe of observation, and the geographic locations where data was collected. Additional information about timeframes of observation and a full list of included countries can be found in Supplementary Tables S1 and S2. A commented R script is available for further insights and replication at https://osf.io/xhe6c.

#### 2.1.1 Data

##### 2.1.1.1 Socialization Goal Norms

The World Value Survey (WVS; Inglehart et al. 2022) contains individual‐level data from representative samples of people aged 16 years and older collected across multiple study waves, each spanning approximately 5 years. We used data from six study waves (1989–1993 until 2017–2022; see Supplementary Table S2). Included study waves contained answers of 2,400 to 40,230 people per country (*M* = 14,336, *SD* = 5,741). The data set contains 51% men and 48% women (1% no information or other gender). The average age of participants was 41.36 years (*SD* = 16.29) of whom 68% were parents. As the data set covers many countries, it includes people from 908 ethnic groups. The largest groups with 2% among the total sample each are South African Black, US White non‐Hispanic, Japanese East Asian Chinese, and Canadian Caucasian/White. Of the WVS participants, 16% had a university degree, while 51% did not (33% no information). The data has been frequently used for cross‐cultural studies (e.g., Park et al. 2014; Santos et al. 2017). We focused on the following WVS item: ‘Here is a list of qualities that children can be encouraged to learn at home. Which, if any, do you consider to be especially important?’ Participants were asked to select up to five socialization goals: imagination, independence, feeling of responsibility, determination and perseverance, religious faith, obedience, hard work, tolerance and respect for other people, thrift saving money and things, and unselfishness. However, the maximum number of selectable socialization goals was not consistent. Therefore, the average number of selected socialization goals was statistically taken into account for regression analyses. For all socialization goals, the proportion of participants selecting a given socialization goal was calculated for each country and study wave as an indicator for the descriptive socialization goal norm (i.e., the relative socialization goal importance). Participant weights provided by the WVS were applied to obtain representative estimates on country level.

##### 2.1.1.2 Child and Adolescent Anxiety Disorder Incidence Rate

The Global Burden of Disease study (GBD; Institute for Health Metrics and Evaluation (IHME) 2020) contains more than 350 country‐level health outcomes and risk factors. The GBD does not conduct own epidemiological surveys but rather draws on administrative records, censuses, clinical trials, demographic surveillance, disease registries, surveys, and vital registration. Standardized estimates are modeled on the basis of available publications (for detailed methods see Ferrari et al. 2024). Anxiety among children and adolescents was measured as the anxiety disorder incidence rate per 100,000 inhabitants in the age group of people 0–19 years old (see Patton et al. 2016). To increase reliability and match the GBD data with the WVS data, the yearly estimates were summarized as mean incidence rates for the 5‐year study waves of the WVS (for details, see Supplementary Table S2).

##### 2.1.1.3 Social and Medical Improvements

The Human Development Report (HDR; UNDP (United Nations Development Programme), 2022) provides global information on country‐level variables that are linked to overall societal wellbeing. As a control variable for this study, the human development index was selected from the HDR. It captures various aspects of social and medical factors (e.g., gross national income, average years of schooling, life expectancy) into a single measure and is available for numerous countries across the globe. Previous research found associations between the human development index and socialization goal norms (Bond and Lun 2014) as well as socioemotional development of children and adolescents (Bornstein et al. 2021). Again, the yearly estimates were summarized as mean values for the 5‐year study waves of the WVS (for details, see Supplementary Table S2).

#### 2.1.2 Data Analyses

Data was retrieved from the websites of the WVS (https://worldvaluessurvey.org), GBD (https://vizhub.healthdata.org/gbd‐results), and HDR (https://hdr.undp.org) in August 2023. WVS, GBD, and HDR data was merged. Study waves with missing data were removed per country. Data was included only for countries with full information for at least two study waves (i.e., longitudinal data). The final data set comprised data of 70 countries across six study waves. On average 3.4 study waves were included per country (*SD* = 1.3). The data contained meaningful temporal variation in the study measures (Supplementary Table S4).

##### 2.1.2.1 Cultural Dimensions

A principal component analysis (PCA) was conducted on the socialization goal norm indicators to reduce dimensionality. PCA transforms correlated variables into uncorrelated principal components (PCs), with each PC representing a weighted combination of the original variables. For all variables, loadings on each PC are calculated. PCA offers two key advantages: it reveals broader socialization goal dimensions and reduces issues with multicollinearity in subsequent regression analyses. The optimal number of PCs was decided based on parallel analysis and eigenvalues of principal components. PCs and variable loadings were computed based on this decision. Additionally, separate PCAs were conducted for each study wave to test for temporal invariance of the correlational structure among socialization goal norm indicators. In addition to incorporating the PCs in our following analyses, we also included the ten socialization goal norm variables.

##### 2.1.2.2 Associations Between Socialization Goal Norms and Anxiety Disorder Incidence Rate for Children and Adolescents on Country Level

Following, we tested the country‐level hypothesis concerning cross‐temporal associations between socialization goal norms and the anxiety disorder incidence rate. A cross‐temporal analysis can deliver correlational insights into the question whether a pattern of change in one variable related to a pattern of change in another variable. Is an increase in the importance of certain socialization goals over time linked to an increase or decrease in the anxiety disorder incidence rate? All regression analyses accounted for the longitudinal structure of the data using linear mixed‐effects models (i.e., random intercepts for countries; Rosseel 2012). The advantage of this cross‐temporal regression approach compared to other methods (e.g., bivariate latent change score models) is that the number of study waves per country can be flexible. Countries with less than six measurement occasions can still be included. This allows for the investigation of a large sample covering many countries.

In a first step, we conducted bivariate associations between anxiety disorder incidence rate and socialization goal norm PCs as well as specific socialization goal norms across all countries. In a second step, we tested the same associations while statistically controlling for cross‐temporal associations between anxiety disorder incidence rate and the human development index. In a third step, we again tested the same association but separately for WEIRD and non‐WEIRD countries. European and Anglophone (i.e., Western) countries that are ranked as *high‐income* countries by the World Bank (The World Bank 2025) and rated as *democracies* in the V‐Dem Project (Lührmann et al. 2018) were considered WEIRD for this analysis. Given the potential ambiguity of the WEIRD vs. non‐WEIRD distinction, we also compared Western vs. non‐Western and high‐ vs. low‐income countries (see Supplementary Table S1).

Next, stepwise multiple regression analysis was performed on the anxiety disorder incidence rate. Four prediction models were compared: a null model without fixed effects (M0), a model with PCs as predictors (M1), a model with PCs and the human development index as predictors (M2), and a model with PCs and the human development index as predictors that accounted for differences between WEIRD and non‐WEIRD countries (i.e., interaction terms; M3).

Finally, another stepwise multiple regression was performed on the anxiety disorder incidence rate to test the fixed effects of the endorsement of specific socialization goals rather than the broad socialization goal dimension earlier. Based on the strength of the bivariate association with the outcome, specific socialization goal norms were added one after another into a linear mixed‐effects model predicting anxiety disorder incidence rates while controlling for effects of the human development index. The analysis concluded when adding another predictor no longer increased the explained variance. Restricted maximum likelihood was used to compute model estimates while maximum likelihood was used for model comparisons.

### 2.2 Results

#### 2.2.1 Descriptive Results

Mean, standard deviation, and range of all study variables summarized across study waves are depicted in Table 1. The anxiety disorder incidence per 100,000 inhabitants showed large cross‐country differences ranging from 301.63 cases on average in Kazakhstan to 1,157.88 cases on average in Norway (*M* = 649.72, *SD* = 231.90). The overall most frequently selected socialization goals were feeling of responsibility (*M* = 0.70, *SD* = 0.12) as well as tolerance and respect for other people (*M* = 0.69, *SD* = 0.11). The overall least frequently selected socialization goals were imagination (*M* = 0.22, *SD* = 0.09) as well as unselfishness (*M* = 0.31, *SD* = 0.10).

**TABLE 1:** Descriptive statistics summarized on country level.

Variable	*M*	*SD*	*Min*	*Max*	*φ_PC1_ (SD)*	*φ_PC2_ (SD)*
Anxiety disorder incidence per 100k	649.72	231.90	301.63 (Kazakhstan)	1157.88 (Norway)	—	—
Principal components of socialization goals						
PC1 (independence‐interdependence)	−0.04	0.96	−1.93 (Ghana)	2.10 (Japan)	—	—
PC2 (civility‐practicality)	−0.02	0.94	−1.64 (Republic of Korea)	2.13 (Norway)	—	—
Specific Socialization goals						
Imagination	0.22	0.09	0.06 (Trinidad and Tobago)	0.46 (Norway)	0.59 (0.36)	0.48 (0.26)
Independence	0.48	0.15	0.24 (Pakistan)	0.90 (Norway)	0.70 (0.09)	0.13 (0.28)
Feeling of responsibility	0.70	0.12	0.40 (Ghana)	0.91 (Norway)	0.55 (0.15)	0.27 (0.35)
Determination perseverance	0.38	0.11	0.15 (Egypt)	0.65 (Slovenia)	0.70 (0.29)	−0.14 (0.34)
Religious faith	0.39	0.25	0.02 (China)	0.87 (Indonesia)	−0.83 (0.29)	0.00 (0.28)
Obedience	0.39	0.17	0.06 (Japan)	0.78 (Ghana)	−0.82 (0.26)	0.17 (0.41)
Hard work	0.56	0.21	0.08 (Sweden)	0.91 (Georgia)	−0.25 (0.30)	−0.77 (0.31)
Tolerance and respect for other people	0.69	0.11	0.41 (Ethiopia)	0.91 (Sweden)	0.14 (0.34)	0.74 (0.33)
Thrift saving money and things	0.36	0.12	0.14 (Norway)	0.62 (Republic of Korea)	0.48 (0.51)	−0.58 (39)
Unselfishness	0.31	0.10	0.06 (Germany)	0.51 (Rwanda)	−0.12 (0.20)	0.37 (0.50)
Human development index	0.74	0.12	0.43 (Ethiopia)	0.92 (Netherlands)	—	—

*Note*: *N*_country_ = 70 (used for *M*, *SD*, *Min*, *Max*); *M*, mean; *Max*, maximum; *Min*, minimum; *SD*, standard deviation.

#### 2.2.2 Cultural Dimensions

**FIGURE 1:**
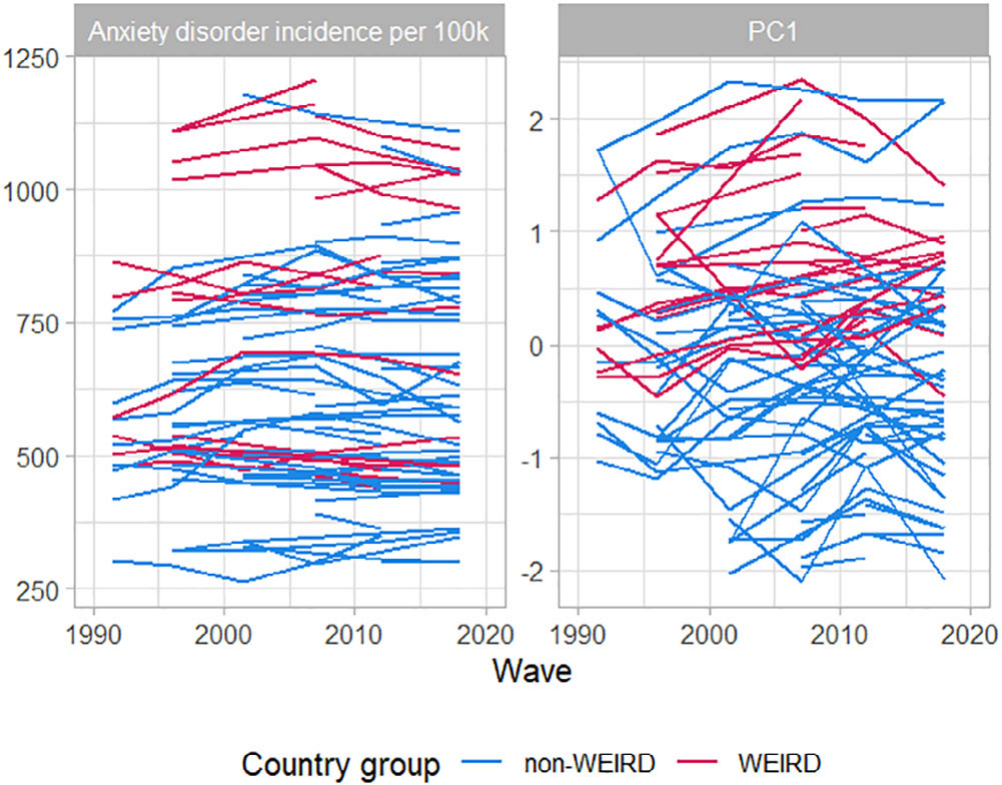
Temporal changes in anxiety incidence rate and socialization goal norms (principal component PC1).

Parallel analysis suggested two PCs to describe socialization goal norms. Three principal components had eigenvalues > 1. Because the third PC had an eigenvalue close to 1 (*λ*_3_ = 1.18), PCA was performed specifying two PCs. PC1 (62% explained variance) was characterized by a high emphasis on independence (*φ* = 0.70) and low emphasis on religious faith (*φ* = −0.83) and obedience (*φ* = −0.82). Therefore, PC1 was labeled *independence‐interdependence*. PC2 (38% explained variance) was characterized by a high emphasis on tolerance and respect for other people (*φ* = 0.74) and low emphasis on hard work (*φ* = −0.77). Therefore, PC2 was labeled *civility‐practicality* (for labelling see Bond and Lun 2014). The loadings of all ten socialization goal variables on the PCs are depicted in Table 1. The subsequent PCAs per study wave showed some instability of the intercorrelation structure across time. For the first principal component PC1, this was less problematic (except for some deviations in the second study wave). However, loadings on the second principal component PC2 showed substantial variation. Thus, we focused on PC1. See Table 1 for standard deviation of loadings and Supplementary Figure S1 for further details. Temporal changes in PC1 alongside changes in the anxiety disorder incidence rate are portrayed in Figure 1.

#### 2.2.3 Associations between Socialization Goal Norms and Anxiety Disorder Incidence Rate for Children and Adolescents on Country Level

##### 2.2.3.1 Bivariate Associations

**FIGURE 2:**
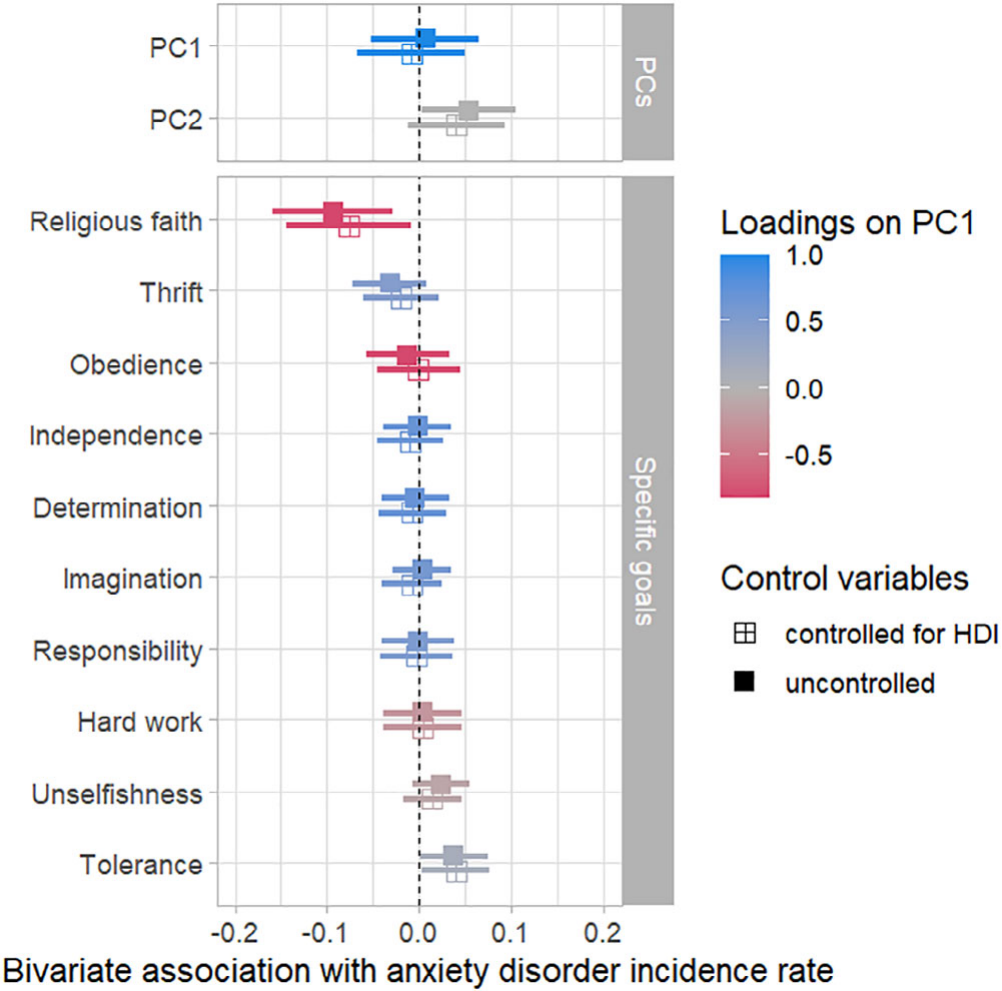
Bivariate cross‐temporal correlations with and without statistical control for changes in the human development index (HDI).

Bivariate association between anxiety disorder incidence rate and socialization goal norms are presented in Figure 2. PC1 (independence‐interdependence) was not correlated with anxiety disorder incidence rate (*r* = 0.01, *p* = 0.812). PC2 was positively correlated with anxiety disorder incidence rate. A greater emphasis on civility (vs. practicality) was linked with higher anxiety disorder incidence rates (*r* = 0.05, *p* = 0.040). Among the specific socialization goal norms, only two meaningful correlations emerged. A greater emphasis on religious faith was linked with lower anxiety disorder incidence rate (*r* = −0.09, *p* = 0.006). A greater emphasis on tolerance and respect for other people was linked to higher anxiety disorder incidence rate (*r* = 0.04, *p* = 0.044). The estimates did not substantially change when statistically controlling for the human development index as a measure of overall societal development. See Supplementary Table S3 for coefficients.

**FIGURE 3:**
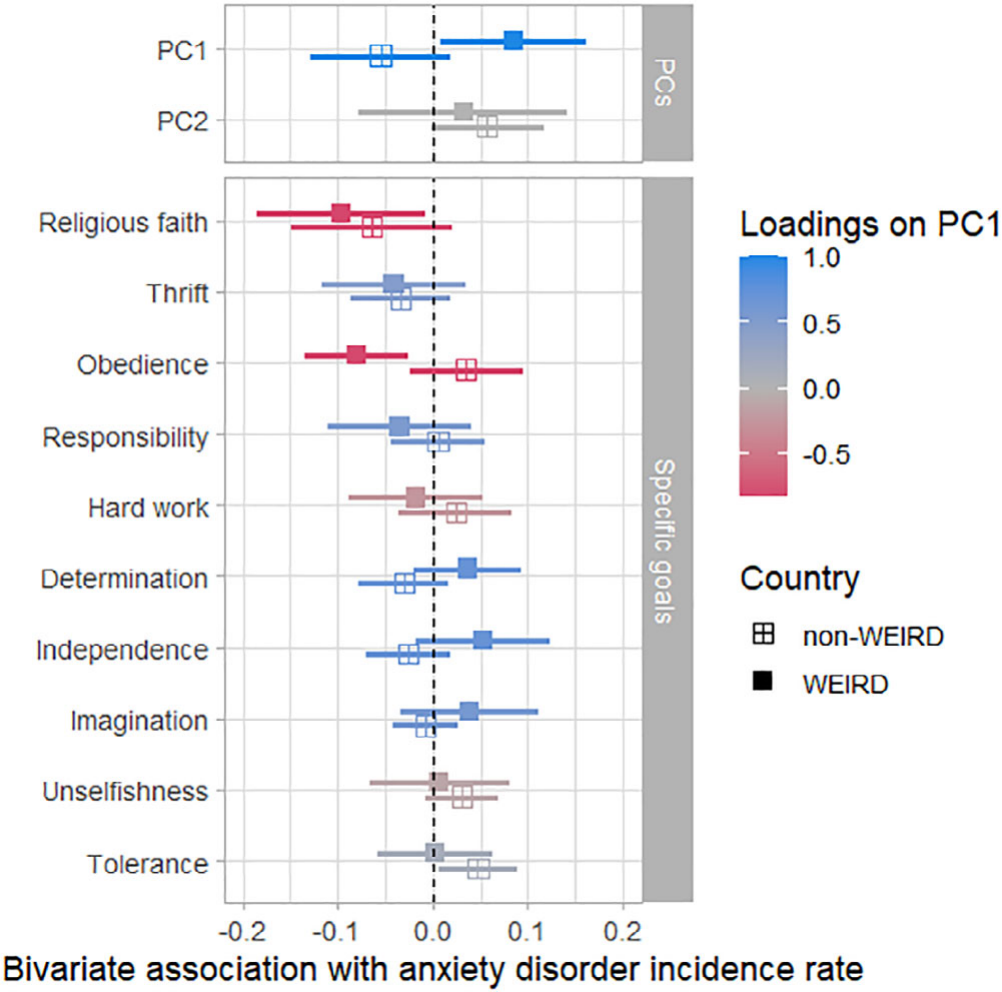
Bivariate cross‐temporal correlations for WEIRD and non‐WEIRD countries.

Figure 3 shows the same bivariate associations separately for WEIRD (*n* = 21) and non‐WEIRD (*n* = 49) countries. It revealed a pattern that socialization goal norms connected to independence (vs. interdependence) tended to be more positively associated with anxiety disorder incidence rate in WEIRD countries (i.e., more anxiety) while more negatively associated with anxiety disorder incidence rate in non‐WEIRD countries (i.e., less anxiety). The PC independence‐interdependence was positively associated with more anxiety across time in WEIRD countries only (*r* = 0.09, *p* = 0.035). The same pattern emerged when comparing Western and non‐Western countries as well high‐come and not high‐income countries. Rising importance of independence‐related socialization goals was only linked to declining mental health in Western (*r* = 0.07, *p* = 0.038) and high‐income countries (*r* = 0.09, *p* = 0.047). Exploring specific socialization goal norms in WEIRD countries, negative correlations emerged between anxiety disorder incidence rate and religious faith (*r* = −0.10, *p* = 0.038) as well as obedience (*r* = −0.08, *p* = 0.007). In non‐WEIRD countries, anxiety disorder incidence rate was positively correlated with tolerance and respect for other people (*r* = −0.05, *p* = 0.027). See Supplementary Table S3 for coefficients.

##### 2.2.3.2 Stepwise Multiple Regression Analysis: Principal Components

Table 2 shows the standardized regression coefficients for the linear mixed models predicting anxiety disorder incidence rate from socialization goal norm PCs. The regression model predicting anxiety disorder incidence rate from PCs only (M1) did not explain more variance than a null model without any fixed effects, *R^2^*_fixed effects_ = 0.06, *F* (5, 3) = 4.28, *p* = 0.117. Adding the human development index as a predictor (M2) led to an increase in explained variance compared to the null model, *R^2^*_fixed effects_ = 0.09, *F* (6, 3) = 10.22, *p* = 0.017. The final model which included custom effects for WEIRD and non‐WEIRD countries increased the explained variance further compared to the previous model, *R^2^*_fixed effects_ = 0.19, *F* (10, 6) = 17.07, *p* = 0.002. This best‐fit model included no main effects for the PCs, but for the control variables human development index (*β* = 0.08, *p* = 0.007) and the group difference between WEIRD and non‐WEIRD countries (*β* = 0.69, *p* = 0.010). Interaction effects (i.e., differential effects for WEIRD and non‐WEIRD countries) emerged for PC1 independence‐interdependence (*β* = 0.21, *p* = 0.004) and human development index (*β* = −0.14, *p* = 0.044). Similar to the previous analysis, PC1 independence‐interdependence had a slight negative but insignificant association with anxiety disorder incidence rate in non‐WEIRD countries (reference group) and a more positive association in WEIRD countries.

**TABLE 2:** Standardized regression coefficients: principal components.

Model	M0		M1		M2		M3	
	*β*	*CI*	*β*	*CI*	*β*	*CI*	*β*	*CI*
Intercept	−0.01	[−0.26, 0.24]	−0.01	[−0.25, 0.24]	0.00	[−0.24, 0.23]	−0.22	[−0.49, −0.06]
Fixed effects								
PC1 (independence‐interdependence)	—	—	0.01	[−0.05, 0.07]	−0.01	[−0.07, 0.05]	−0.06	[−0.12, 0.01]
PC2 (civility‐practicality)	—	—	**0.05** *	[0.00, 0.11]	0.04	[−0.01, 0.09]	0.03	[−0.03, 0.09]
HDI (human development index)	—	—	—	—	**0.07** *	[0.01, 0.12]	**0.08** **	[0.02, 0.14]
Group (WEIRD vs. non‐WEIRD)	—	—	—	—	—	—	**0.69** *	[0.17, 1.21]
PC1 × Group	—	—	—	—	—	—	**0.21** **	[0.07, 0.36]
PC2 × Group	—	—	—	—	—	—	−0.01	[−0.13, 0.12]
HDI × Group	—	—	—	—	—	—	**−0.14** *	[−0.27, 0.00]
Random effects: Country								
Residual (σ^2^)	0.02		0.02		0.02		0.02	
Intercept (τ_00_)	1.11		1.05		1.01		0.92	
*R^2^*_fixed effects_	0.00		0.06		0.09		0.19	
*R^2^*_total_	0.98		0.98		0.98		0.98	

*Note*: Non‐WEIRD was set as reference for the variable Group. N _countries_ = 70, N _Observations_ = 236, * *p* < 0.05; ** *p* < 0.01.

##### 2.2.3.3 Stepwise Multiple Regression Analysis: Specific Socialization Goal Norms

Table 3 shows the standardized regression coefficients for the linear mixed models predicting anxiety disorder incidence rate from specific socialization goal norms. The regression model predicting anxiety disorder incidence rate from human development index and the importance of religious faith (M1) explained more variance than a basic model using only the human development index, *R^2^*_fixed effects_ = 0.04, *F* (5, 4) = 4.88, *p* = 0.027. Adding the importance of tolerance and respect for other people (M2) further increased prediction, *R^2^*_fixed effects_ = 0.07, *F* (6, 5) = 5.64, *p* = 0.018. Additionally including the importance of thrift, saving money and things (M3) did not lead to more explained variance, *R^2^*_fixed effects_ = 0.08, *F* (7, 6) = 1.11, *p* = 0.293. The best‐fit model M2 included a negative effect of the importance of religious faith (*β* = −0.08, *p* = 0.016) as well as positive effects of tolerance and respect for other people (*β* = 0.04, *p* = 0.019) and human development index (*β* = 0.06, *p* = 0.022). Across all mixed‐effects models, total explained variance was high (*R^2^*_total_ = 0.98), reflecting large and stable between‐country differences in anxiety disorder incidence captured by the random intercepts. Variance explained by the fixed effects (i.e., within‐country effects) was comparatively small (*R^2^*_fixed effects_ = 0.00–0.19).

**TABLE 3:** Standardized regression coefficients: specific socialization goal norms.

Model	M0		M1		M2		M3	
	*β*	*CI*	*β*	*CI*	*β*	*CI*	*β*	*CI*
Intercept	0.00	[−0.25, 0.24]	0.00	[−0.25, 0.24]	0.00	[−0.24, 0.24]	0.00	[−0.24, 0.24]
Fixed effects								
Human development index	**0.07** **	[0.02, 0.12]	**0.06** *	[0.01, 0.11]	**0.06** *	[0.01, 0.11]	0.05	[0.00, 0.10]
Religious faith	—	—	**−0.08** *	[−0.14, −0.01]	**−0.08** *	[−0.15, −0.02]	**−0.09** *	[−0.16, −0.02]
Tolerance and respect for other people	—	—	—	—	**0.04** *	[0.01, 0.08]	**0.04** *	[0.00, 0.08]
Thrift, saving money and things	—	—	—	—	—	—	−0.02	[−0.06, 0.02]
Random effects: Country								
Residual (σ^2^)	0.02		0.02		0.02		0.02	
Intercept (τ_00_)	1.05		1.07		1.04		1.03	
*R^2^*_fixed effects_	0.06		0.04		0.07		0.08	
*R^2^*_total_	0.98		0.98		0.98		0.98	

*Note*. N _countries_ = 70, N _Observations_ = 236, * p < 0.05; ** p < 0.01.

### 2.3 Discussion

The results of the PCA closely aligned with the factor analysis conducted by Bond and Lun (2014) on a single wave of the World Values Survey (2005–2009). In their study, the authors identified the same two dimensions and labeled them self‐directedness versus other‐directedness and civility versus practicality. We retained the latter but renamed the first as independence versus interdependence to better align with contemporary frameworks of cultural differences. As expected, independence‐interdependence accounted for a substantial proportion of variance across countries and time points, supporting its central role in the present research. Civility‐practicality seemed to be an instable construct across time points and results regarding this dimension should be interpreted with caution. The Supplementary Discussion provides an interpretation of between‐country differences.

Globally, there was no link between independence‐interdependence and anxiety in children and adolescents. Rather, the relationship between changes in independence‐related socialization norms and anxiety depended on the country. In non‐WEIRD countries, the endorsement of independence goals was unrelated to child and adolescent anxiety. The interaction effect showed that this was different for WEIRD countries. There, independence goals were more likely to be linked with adverse mental health effects. While we hoped to generalize findings of previous research to the global stage, this WEIRD‐specific result aligns with the literature reviews focusing on WEIRD countries only (Humphrey and Bliuc 2021). The cultural fraud hypothesis (Eckersley 2006) offers a potential explanation. The sample of non‐WEIRD countries included many emerging and developing countries. In these societies that are typically characterized by stronger interdependence‐related values, a shift towards more independence may drive the same economic growth that Eckersley (2006) believed to have happened decades ago in WEIRD countries. More independence norms may help economic development by facilitating innovation, competition, and entrepreneurship. This may increase quality of life and therefore diminish anxiety disorders in children and adolescents. There may be an optimal match between independence norms and economic development state that best supports children's and adolescent's mental health. More research is needed to confirm this hypothesis.

In our search for globally relevant cultural factors, we identified religious faith and tolerance‐related socialization goals as meaningful correlates. However, the latter was less relevant to this analysis, as tolerance does not directly align with the cultural dimension of independence‐interdependence (PC1). Moreover, the observed positive association between greater tolerance and higher anxiety disorder rates may reflect a bias in health data, where intolerance and stigma suppress reported anxiety incidence (Schomerus and Angermeyer 2008).

The small negative correlation between changes in the importance of religious faith as a quality in children and changes in the anxiety disorder incidence is an indication for the potential link between religion as a specific aspect of an interdependent social orientation and child and adolescent mental health. Religious socialization may serve as a protective factor against anxiety problems for children and adolescents. A recent systematic review and meta‐analysis of longitudinal studies support the present finding of a (potentially directed) relationship from spiritual wellbeing to child and adolescent mental health (Aggarwal et al. 2023). Religious socialization may cultivate a personal sense of hope and purpose in life, while also enhancing prosocial, community‐oriented attitudes and behaviors during childhood and adolescence (Eisenberg et al. 2011).

Given the small effect sizes with confidence intervals indicating potential effect being close to zero (see Tables 2 and 3) and the data‐driven approach used in analyzing specific socialization goals, replicating the link between religiosity norms and early mental health in independent samples is necessary. Additionally, the underlying mechanisms of this association require further investigation. How do societal norms compare to parental values in shaping developmental outcomes at the individual level? And what is the direction of the relationship between religiosity and mental health of children and adolescents?

## 3 Study 2

In the second study, we conducted two follow‐up analyses to examine the micro‐level processes that could underlie the cultural change processes observed in Study 1. Rather than using country‐level data, this study relied on individual‐level data, which allowed us to investigate how cultural norms become embedded in individual beliefs and family practices, and to gain further insight into the processes producing the previous results. As the socially shared importance of religious faith as an important interdependence‐related quality in children turned out to be the strongest predictor and most stable across WEIRD and non‐WEIRD countries, Study 2 investigated individual‐level links between religiosity norms, maternal religiosity, and anxiety symptoms in children and adolescents. Based on Study 2 and causal reasoning presented by Eckersley (2006), we expected a directed link from reductions in religiosity norms to increases in anxiety symptoms across time. This approach allowed us to connect macro‐level cultural shifts with individual‐level experiences, offering a comprehensive perspective on cultural change. This design captured relevant time period effects of cultural change as well as age effect. Cohort effects were eliminated as data came from a single birth cohort.

### 3.1 Methods

The follow‐up analyses utilized another publicly available individual‐level data on religiosity and anxiety symptoms in children and adolescents. The data set was selected on the basis of the same criteria as in Study 1. A commented R script is available for further insights and replication at https://osf.io/xhe6c.

#### 3.1.1 Data

The Future of Families and Child Wellbeing Study (FFCWS; Reichman et al. 2001) is a longitudinal study tracking 4,898 children born in large U.S. cities between 1998 and 2000 until their early adulthood, along with their families. This analysis focused on maternal reports from study waves conducted when the participating children or adolescents were 3, 5, 9, and 15 years old. From the total, 52% of children and adolescents were male, 48% female. At birth, mothers were on average 25.28 years old (*SD* = 6.04). Of the mothers ,47% identified as Black non‐Hispanic, 27% Hispanic, 21% White non‐Hispanic, 4% other race, and race/ethnicity information was missing for 5%. Of the mothers, 35% had a university degree at the time of their child's birth, while 65% did not. We examined responses to the items: “My religious faith is a guide for the way I treat my family / for my daily life” and “My child is too fearful or anxious.” Responses were recorded on a 3‐ or 4‐point Likert scale. Notably, only at the 3‐year study wave, the religion‐related item specifically referenced family. Therefore, we conducted separate analyses (i.e., including the 3‐year wave and excluding it). All items were recoded so that higher values depicted stronger agreement with the item. In line with the other analyses, we also calculated the descriptive norm of religious faith using the sample mean per study wave.

#### 3.1.2 Data Analyses

Data was retrieved from the FFCWS website (https://ffcws.princeton.edu/) in November 2024. For the multiple analyses using the FFCWS individual‐level data, between 2,306 and 3,731 U.S. children were included.

##### 3.1.2.1 Associations Between Religiosity (Norms) and Anxiety Symptoms in Children and Adolescents on Individual Level

Based on the results of the preceding analyses, we were interested in the role of religious faith for child and adolescent anxiety symptoms. We replicated the same cross‐temporal analysis in an independent data set using a slightly different approach. Here, the level of analysis was no longer country but child/adolescent. While we previously compared all people 0–19 years of age in a certain country in the early 1990s to other people 0–19 years of age in the late 2010s in this country, we now compare a child at age 3 to the same adolescent at age 15.

Stepwise multiple regression analysis was performed on the maternal ratings of child and adolescent anxiety symptoms. Three prediction models were compared: a null model without fixed effects (M0), a model with maternal religiosity as a predictor (M1), a model with maternal religiosity and the religiosity norm (i.e., sample mean per wave) as predictors (M2). We conducted separate analyses including the 3‐year wave and excluding it because of the difference in the religiosity item. Again, restricted maximum likelihood was used to compute model estimates while maximum likelihood was used for model comparisons. Additional exploratory analyses were conducted, controlling these associations for family‐ and sample‐level temporal variation in social, economic, and medical indicators (i.e., receipt of social welfare, household income, and unmet healthcare needs).

##### 3.1.2.2 Directed Links Between Religiosity and Anxiety Symptoms in Children and Adolescents on Individual Level

Since the nature of the conducted correlational analyses does not allow for causal interpretation, we aimed to calculate cross‐lagged panel models (CLPMs). Besides autoregressive *a* paths, they model paths from earlier measures of one trait to later measures of another trait, referred to as causal *c* paths. CLPMs assess how past levels of one trait can predict later levels of another. This is a precondition for causality and may help to falsify causal claims. Yet, such temporal relationships may also be influenced by unobserved third variables (Usami et al. 2019). A CLPM was not feasible for the country‐level data since not all countries were included in every wave, resulting in an insufficient sample size. Consequently, we conducted the CLPM using only the individual‐level data. However, this design does not allow for the inclusion of religiosity norms, as the group mean is identical across all cases, leaving no variance to analyze. We modelled the *a* and *c* paths from maternal ratings of individual religiosity and anxiety symptoms at 3 years to the same maternal ratings when the adolescent was 15 years old (Rosseel 2012). The CLPM was repeated controlling for individual‐level social, economic, and medical indicators using residualized variables.

### 3.2 Results

#### 3.2.1 Associations between Religiosity (Norms) and Anxiety Symptoms in Children and Adolescents on Individual Level

Table 4 shows the standardized regression coefficients for the linear mixed models predicting individual anxiety symptoms from religiosity on individual level. Initially, we included all four study waves containing slightly different religiosity items. The regression model predicting anxiety symptoms from maternal religiosity only (M1) explained more variance than a null model without any fixed effects, *R^2^*_fixed effects_ = 0.00, *F* (4, 3) = 8.22, *p* = 0.004. Adding the descriptive religiosity norm (i.e., the sample mean per wave; M2) increased the prediction, *R^2^*_fixed effects_ = 0.01, *F* (5, 4) = 62.03, *p* < 0.001. This best‐fit model included a negative effect of maternal religiosity (*β* = −0.02, *p* = 0.030) and a stronger negative effect of religiosity norm (*β* = −0.07, *p* < 0.001). A corresponding analysis using only the three study waves with matching item wordings yielded the same pattern of results. M2 showed the best model fit. Coefficients were largely similar (Table 4). Similar to Study 1, the total explained variance was modest (*R^2^*_total_ = 0.16–0.18) and was primarily driven by stable between‐family differences in anxiety symptoms captured by the random intercepts. In contrast, variance attributable to the fixed effects (i.e., within‐family associations over time) was very small (*R^2^*_fixed effects_ = 0.00–0.01). The link between maternal religiosity and mental health sustained when controlling for social, economic, and medical indicators. Similarly, religiosity norm remained a meaningful predictor when controlling for sample‐level indicators. However, these coefficients should be interpreted with caution because of multicollinearity issues (Supplementary Table S5).

**TABLE 4:** Standardized regression coefficients: child‐level analysis.

	Three study waves (5–15 years old)						Four study waves (3–15 years old)					
Model	M0		M1		M2		M0		M1		M2	
	*β*	*CI*	*β*	*CI*	*β*	*CI*	*β*	*CI*	*β*	*CI*	*β*	*CI*
Intercept	0.00	[−0.02, 0.03]	0.00	[−0.02, 0.03]	0.00	[−0.02, 0.03]	0.00	[−0.02, 0.02]	0.00	[−0.02, 0.02]	0.00	[−0.02, 0.02]
Fixed effects												
Maternal religiosity	—	—	−0.03**	[−0.05, −0.01]	**−0.0** **2** *	[−0.05, 0.00]	—	—	**−0.03** **	[−0.05, −0.01]	**−0.02** *	[−0.04, 0.00]
Religiosity norm	—	—	—	—	**−0.06** ***	[−0.08, −0.04]	—	—	—	—	**−0.07** ***	[−0.08, −0.05]
Random effects: Child												
Residual (σ^2^)	0.83		0.83		0.82		0.84		0.84		0.83	
Intercept (τ_00_)	0.17		0.17		0.17		0.17		0.16		0.17	
*R^2^*_fixed effects_	0.00		0.00		0.00		0.00		0.00		0.01	
*R^2^*_total_	0.17		0.17		0.18		0.16		0.17		0.17	

*Note*: Three waves: N _children_ = 3274, N _Observations_ = 8776; Four waves: N _children_ = 3731, N _Observations_ = 12,131; * *p* < 0.05; ** *p* < 0.01; *** *p* < 0.001.

#### 3.2.2 Directed Links between Religiosity and Anxiety Symptoms in Children and Adolescents on Individual Level

In total, *N* = 2,306 children and adolescents were included in the CLPM analysis. The model revealed meaningful *a* paths for maternal religiosity (*β* = 0.40, *p* < 0.001) and anxiety symptoms (*β* = 0.11, *p* < 0.001). A *c* path emerged from maternal religiosity at 3 years to adolescent anxiety symptoms at 15 years (*β* = −0.05, *p* = 0.011). No *c* path was found from anxiety symptoms at 3 years to maternal religiosity at 15 years (*β* = 0.00, *p* = 0.824). Controlling for social, economic, and medical indicators did not change the results (Supplementary Table S7).

### 3.3 Discussion

Study 2 successfully replicated the link between religiosity and anxiety in children and adolescents which emerged in Study 1. In this independent sample, similar cross‐temporal effect sizes were found. In Study 1, effects ranged between −0.08 and −0.09. In Study 2, effects ranged between −0.06 and −0.07.

The religiosity norm (as captured by a simple group mean) was a stronger predictor for individual child and adolescent symptoms than the religiosity of their own mother. This reinforces the focus on socially shared norms. While caregivers are very important for human development (e.g., Keller and Kärtner 2013), children and adolescents learn a lot outside their home. For example, recent research has pointed towards schools as important places of cultural reproduction (Fong et al. 2024). In the specific case of religiosity norms, shared religious beliefs may provide children and adolescents with a structured framework for understanding the world, offering clear guidelines on behavior, morality, and decision‐making. In a complex modern world, these rules may create a sense of stability and security, reducing uncertainty and anxiety. Additionally, shared religious beliefs may foster a strong sense of belonging within communities, reinforcing social support beyond the immediate family.

While CLPMs cannot establish causality, the missing longitudinal effect from anxiety to religiosity suggest either religiosity affects anxiety or there is a third variable driving the observed correlations. The effectiveness of mental health interventions involving religious practices and spirituality (e.g., prayer) points towards a causal effect of religiosity on child and adolescent mental health. On the other hand, feelings of being abandoned by or blaming God (i.e., negative religious coping) was found to have a negative correlation with mental health among children and adolescents (Aggarwal et al. 2023).

## 4 General Discussion

The presented studies aimed to shed light on the potential consequences of cultural change for child and adolescent mental health with a specific focus on socialization goals as an aspect of culture that directly focuses on children and adolescents. Based on the cultural fraud hypothesis (Eckersley 2006) and empirical evidence from WEIRD countries (Humphrey and Bliuc 2021), we expected to find associations between socialization goals linked to an independent (vs. interdependent) social orientation and an increased incidence rate of anxiety disorders among children and adolescents worldwide. The results indicated only limited evidence for this hypothesis.

This longitudinal analysis of socialization goals and anxiety in children and adolescents across three decades and 70 countries worldwide, revealed five key findings. First, global changes in independence‐interdependence as a broad socialization goal dimension (i.e., principal component) were not related to changes in the anxiety disorder incidence rate. Second, this association seemed to differ between WEIRD and non‐WEIRD countries. Independence‐focused socialization norms had more adverse effects in WEIRD countries compared to non‐WEIRD countries. Third, changes in the importance of religious faith as well as tolerance and respect for other people were linked to changes in the anxiety disorder incidence rate across countries, whereas religious faith stood out as the strongest predictor. A decrease in the endorsement of religious faith as an important quality in children went along with an increase in the number of children and adolescents diagnosed with anxiety disorders. When tolerance and respect for other people became less important as a quality in children, less children and adolescents were diagnosed with anxiety disorders. Fourth, the religiosity norm appeared to have a stronger association with individual child and adolescent anxiety symptoms than maternal religiosity. Fifth, there was evidence for a directed relationship from religiosity to child and adolescent anxiety but not for the other direction. Societies that are becoming increasingly secular should take this into account and support children and adolescents in developing a sense of purpose and belonging without religion.

Religiosity may not be specifically linked to factors in the etiology of anxiety disorders. Rather, religious socialization norms may help children and adolescents to build resilience by increasing their mental health. The Ryff six‐factor model of mental well‐being (Ryff and Keyes 1995) outlines key dimensions that contribute to mental health. Autonomy reflects a person's ability to maintain independence and self‐regulation despite social pressures. Environmental mastery refers to effectively managing life's challenges and creating opportunities to meet personal needs. Personal growth emphasizes openness to new experiences and continuous self‐improvement. Positive relations with others highlight the importance of meaningful social connections. Purpose in life captures having clear goals and a sense of direction, while self‐acceptance reflects a positive self‐view and recognition of one's strengths and limitations.

According to the model, cultural norms that encourage children's and adolescent's religiosity can positively influence several aspects of mental health. While autonomy emphasizes independent decision making, a structured belief system can still support it by providing a framework that helps young people confidently maintain their opinions (Błażek and Besta 2012). Religion also fosters environmental mastery by offering routines, rituals, and coping strategies that help children and adolescents navigate new situations (Selman and Dilworth‐Bart 2024). Many religious teachings encourage self‐reflection and moral development, supporting personal growth (Dollahite and Marks 2019). Additionally, religiosity promotes social cohesion, strengthening positive relationships by fostering a sense of belonging (ten Kate et al. 2017). A religious upbringing can also contribute to a strong sense of purpose, providing children and adolescents with meaning and direction in life (Tirri and Quinn 2010). Lastly, narratives centered on forgiveness, love, and self‐worth can enhance self‐acceptance (Dollahite and Marks 2019). Altogether, these factors illustrate how societal expectations around religiosity can align with the model of mental well‐being to explain the protective effects of “religious faith” norm on child and adolescent anxieties.

In modern times when religion is losing its importance worldwide (Inglehart 2021), more and more children and adolescents may suffer from mental health problems (Twenge et al. 2019). To address this, societies should invest in alternative sources of social cohesion that provide similar psychological and developmental benefits. Schools and non‐governmental organizations could play a role in fostering meaningful connections by promoting collective values, rituals, and mentorship. Expanding access to structured extracurricular activities, such as sports teams, arts programs, and youth groups, can help children and adolescents build identity and resilience. Additionally, policymakers should prioritize family‐friendly policies that strengthen social support networks, ensuring that children and adolescents have stable environments in which to grow up. By filling the void that is left by the vanishing of religious norms, societies may mitigate the psychological costs of secularization and promote children's and adolescent's mental health.

### 4.1 Limitations and Future Studies

To connect culture, mental health, and human development globally, three data sets had to be combined (Study 1). The different data collection procedures and periods led to slight differences in the included years for some study waves. The WVS gradually expended with regards to the included country samples over the years of its existence. Some countries were only included in some waves and later dropped out. This led to a varying amount of available data points per country and hence reduced statistical power and generalizability. While the WVS contains data from representative samples collected in various countries, the GBD data set relies on published health data. This may cause biases since health estimates could be more or less reliable in some countries. Furthermore, while our cross‐temporal design reduces concerns about static cross‐national differences in diagnostic norms, it cannot rule out that changing cultural expectations and conceptions of pathology (e.g., concept creep in mental health; Haslam 2016) contributed to the observed within‐country trends. The selection of the human development index was based on previous literature (Bond and Lun 2014) but should be extended to by a range of other relevant variables in future studies (e.g., wealth inequality). However, many predictor variables and a relatively small sample size (since country was the level of analysis) may lead to a lack of power. Future studies need to resolve this issue by finding balance between inclusion of all necessary predictors of child and adolescent mental health and sufficient statistical power. The comparison between WEIRD and non‐WEIRD countries was adapted from previous research that explicitly focused on WEIRD cultures (Eckersley 2006; Humphrey and Bliuc 2021). It is important to note that WEIRD and non‐WEIRD cultures are not homogenous. Future pre‐registered research should use longitudinal, individual‐level data from various countries worldwide to test country‐specific effects of cultural change on child and adolescent mental health. Furthermore, the mechanisms through which cultural norms shape human development should be systematically examined, considering the diverse agents of cultural transmission, such as parents, grandparents, teachers, neighbors, peers, or (social) media figures. The detected effect sizes were generally small, and their real‐world impact remains to be determined. As Baumeister and Lau (2024) emphasize, psychological phenomena rarely have single causes, so additional mechanisms may contribute to changes in child mental health across time; further research is needed to examine these possibilities.

## 5 Conclusion

Using longitudinal data from across the globe, this study unraveled novel links between changes in socially shared socialization goals and changes in child and adolescent mental health. The hypothesis that socialization that increasingly fosters an independent (vs. interdependent) social orientation would be linked to more anxiety problems in children and adolescents could not be fully supported. Instead, this may only be true for WEIRD countries. Across all countries, religious faith as a specific aspect of the interdependent social orientation turned out to be important for child and adolescent mental health. Religious socialization can provide psychological benefits like purpose and positive social relations. As societies become more secular, research should explore alternative modes to support children's and adolescent's development of purpose and belonging without religion. This may inform policymakers with critical evidence to better prevent mental health problems among children and adolescents worldwide.

## Author Contributions

**Leonard Konstantin Kulisch**: designed research, analyzed data, wrote the paper. **Ana Lorena Domínguez Rojas**: wrote the paper. **Silvia Schneider**: wrote the paper. **Babett Voigt**: designed research, wrote the paper.

## Funding

Funded by the Deutsche Forschungsgemeinschaft (DFG, German Research Foundation)—Projektnummer GRK 274877981. Research reported in this publication was supported by the Eunice Kennedy Shriver National Institute of Child Health and Human Development (NICHD) of the National Institutes of Health under award numbers R01HD036916, R01HD039135, and R01HD040421, as well as a consortium of private foundations. The content is solely the responsibility of the authors and does not necessarily represent the official views of the National Institutes of Health.

## Ethics Statement

No ethical approved was needed for this study.

## Conflicts of Interest

The authors declare no conflict of interest.

## Supporting information




**Supporting File 1**: desc70157‐sup‐0001‐SupMat.docx

## Data Availability

Analysis script available at https://osf.io/xhe6c/. The research data are available at https://worldvaluessurvey.org, https://vizhub.healthdata.org/gbd‐results, https://hdr.undp.org, and https://ffcws.princeton.edu. Preprint available at https://doi.org/10.31234/osf.io/btk5h.
