# Supplementary material for: Global Cultural Change and Anxiety in Children and Adolescents: Analyzing Socialization Goals Over Three Decades in 70 Countries
Source: Dev Sci. 2026 Mar 8;29(3):e70157. doi: 10.1111/desc.70157 (PMC12968521; doi:10.1111/desc.70157)
Supplement: Supplementary file 1 — Supporting File 1: desc70157‐sup‐0001‐SupMat.docx [file DESC-29-e70157-s001.docx]

**- Supplementary Material -**

**Supplementary Tables**

Supplementary Table S1. Countries included in the analysis

| Country | | | |
| --- | --- | --- | --- |
| Albania ^b^  Algeria  Andorra ^abc^  Argentina  Armenia  Australia ^abc^  Bangladesh  Brazil  Bulgaria ^abc^  Canada ^abc^  Chile ^c^  China  Colombia  Cyprus ^abc^  Czechia ^abc^  Ecuador  Egypt  Estonia ^abc^ | Ethiopia  Finland ^abc^  Georgia  Germany ^abc^  Ghana  Guatemala  Hungary ^bc^  India  Indonesia  Iran  Iraq  Japan ^c^  Jordan  Kazakhstan  Kyrgyzstan  Lebanon  Libya  Malaysia | Mexico  Morocco  Netherlands ^abc^  New Zealand ^abc^  Nigeria  Norway ^abc^  Pakistan  Peru  Philippines  Poland ^abc^  Republic of Korea ^c^  Moldova ^b^  Romania ^abc^  Russia ^bc^  Rwanda  Serbia ^b^  Singapore ^c^  Slovakia ^abc^ | Slovenia ^abc^  South Africa  Spain ^abc^  Sweden ^abc^  Switzerland ^abc^  Thailand  Trinidad and Tobago ^c^  Tunisia  Turkey  Ukraine ^b^  United Kingdom ^abc^  USA ^abc^  Uruguay ^c^  Venezuela  Vietnam  Zimbabwe |

Note: ^a^ WEIRD countries, ^b^ Western countries, ^c^ high-income countries.

Supplementary Table S2. Timeframe of study waves (I-VI)

| Dataset | Year | | | | | | | | | | | | | | | | |
| --- | --- | --- | --- | --- | --- | --- | --- | --- | --- | --- | --- | --- | --- | --- | --- | --- | --- |
|  | 1989 | 1990 | 1991 | 1992 | 1993 | 1994 | 1995 | 1996 | 1997 | 1998 | 1999 | 2000 | 2001 | 2002 | 2003 | 2004 | 2005 |
| WVS | I | I | I | I | I | II | II | II | II | II | III | III | III | III | III | III | IV |
| GBD | - | I | I | I | I | II | II | II | II | II | III | III | III | III | III | III | IV |
| HDR | - | I | I | I | I | II | II | II | II | II | III | III | III | III | III | III | IV |

(continuation)

| Dataset | Year | | | | | | | | | | | | | | | | |
| --- | --- | --- | --- | --- | --- | --- | --- | --- | --- | --- | --- | --- | --- | --- | --- | --- | --- |
|  | 2006 | 2007 | 2008 | 2009 | 2010 | 2011 | 2012 | 2013 | 2014 | 2015 | 2016 | 2017 | 2018 | 2019 | 2020 | 2021 | 2022 |
| WVS | IV | IV | IV | IV | V | V | V | V | V | - | - | VI | VI | VI | VI | VI | VI |
| GBD | IV | IV | IV | IV | V | V | V | V | V | - | - | VI | VI | VI | - | - | - |
| HDR | IV | IV | IV | IV | V | V | V | V | V | - | - | VI | VI | VI | VI | VI | - |

Note. *GBD*, Global Burden of Disease study; *HDR*, Human Development Report; *WVS*, world value survey.

Supplementary Table S3. Bivariate associations with anxiety disorder incidence

| Variable | All, no control  *n* = 70 | Controlled for HDI *n* = 70 | WEIRD countries *n* = 21 | Non-WEIRD countries *n* = 49 | Western countries *n* = 27 | Non-Western *n* = 43 | High-income *n* = 29 | Not high-income *n* = 41 |
| --- | --- | --- | --- | --- | --- | --- | --- | --- |
| PC1 (independence-interdependence) | .01 | -.01 | .09 * | -.05 | .07 * | -.05 | .09 * | -.05 |
| PC2 (civility-practicality) | .05 * | .04 | .03 | .06 | .03 | .06 | .02 | .06 * |
| Independence | .00 | -.01 | .05 | -.03 | .04 | -.02 | .04 | -.02 |
| Hard work | .00 | .00 | -.02 | .02 | -.01 | .02 | -.01 | .02 |
| Responsibility | .00 | .00 | -.04 | .01 | -.02 | .00 | -.03 | .01 |
| Imagination | .00 | -.01 | .04 | -.01 | .02 | -.01 | .00 | .00 |
| Tolerance | .04 * | .04 * | .00 | .05 * | .01 | .04 * | .03 | .04 |
| Thrift | -.03 | -.02 | -.04 | -.03 | -.04 | -.03 | -.01 | -.05 * |
| Determination | .00 | -.01 | .04 | -.03 | .03 | -.03 | .03 | -.03 |
| Religious faith | -.09 ** | -.08 * | -.10 * | -.06 | -.09 * | -.06 ** | -.11 ** | -.03 |
| Unselfishness | .02 | .01 | .01 | .03 | .00 | .03 | .03 | .02 |
| Obedience | -.01 | .00 | -.08 ** | .04 | -.07 ** | .04 * | -.08 * | .04 |

Note. HDI, human development index; * p < .05; ** p < .01;

Supplementary Table S4. Linear associations between study variables and year of study wave

| Variable | *r* | *p* |
| --- | --- | --- |
| Anxiety disorder incidence per 100k | .02 | .034* |
| Principal components of socialization goals |  |  |
| PC1 (independence-interdependence) | .06 | .022* |
| PC2 (civility-practicality) | .06 | .052 |
| Specific Socialization goals |  |  |
| Independence | .09 | .043* |
| Hard work | .02 | .540 |
| Responsibility | -.07 | .092 |
| Imagination | .14 | .009** |
| Tolerance | -.03 | .489 |
| Thrift | -.11 | .010* |
| Determination | .03 | .489 |
| Religious faith | -.08 | .005** |
| Unselfishness | .13 | .012* |
| Obedience | -.07 | .081 |
| Human development index | .35 | <.001*** |

Note. Controlled for multiple comparisons within specific socialization goals using FDR; * p < .05; ** p < .01; *** p < .001

Supplementary Table S5. Standardized regression coefficients: child-level analysis controlling for context factors, three study waves (5 to 15 years old)

| Model | M3 |  | M4 |  | M5 |  | M6 |  |
| --- | --- | --- | --- | --- | --- | --- | --- | --- |
|  | *β* | *CI* | *β* | *CI* | *β* | *CI* | *β* | *CI* |
| Intercept | 0.00 | [-0.02, 0.02] | 0.00 | [-0.02, 0.03] | 0.00 | [-0.02, 0.03] | 0.00 | [-0.02, 0.03] |
| Fixed effects: Religiosity |  |  |  |  |  |  |  |  |
| Maternal religiosity | **-.03*** | [-.05, .00] | -.02 | [-.04, .00] | -.02 | [-.04, .00] | -.02 | [-.04, .00] |
| Religiosity norm | - | - | **-.48***** | [-.54, -.42] | **1.42***** | [1.23, 1.62] | **.03**** | [.01, .06] |
| Fixed effects: Context |  |  |  |  |  |  |  |  |
| Household income | **-.03*** | [-.05, -.01] | - | - | - | - | **-** | - |
| Welfare recipient | **.05***** | [.03, .07] | - | - | - | - | - | - |
| Unmet healthcare | **.04***** | [.02,.06] | - | - | **-** | - | - | - |
| Income norm | - | - | **.44***** | [.38, .50] | **-** | - | - | - |
| Welfare norm | - | - | - | - | **1.49***** | [1.30, 1.69] | - | - |
| Healthcare norm | - | - | - | - | **-** | - | **-.18***** | [-.20 -.15] |
| Random effects: Child |  |  |  |  |  |  |  |  |
| Residual (σ²) | 0.83 |  | .79 |  | .79 |  | .79 |  |
| Intercept (τ_00_) | 0.16 |  | 0.19 |  | 0.19 |  | 0.19 |  |
| *R²* _fixed effects_ | .01 |  | .03 |  | .03 |  | .03 |  |
| *R²* _total_ | .17 |  | .21 |  | .21 |  | .21 |  |

Note. Because norm variables are computed as means per study wave, regression models would run into multicollinearity issues when trying to fit various norm variables in a model. Therefore, only one context norm variable is included in each model. Coefficients for norm variables should be interpreted with caution. N _children_ = 3,109, N _Observations_ = 8,143; * p < .05; ** p < .01; *** p < .001.

Supplementary Table S6. Standardized regression coefficients: child-level analysis controlling for context factors, four study waves (3 to 15 years old)

| Model | M3 |  | M4 |  | M5 |  | M6 |  |
| --- | --- | --- | --- | --- | --- | --- | --- | --- |
|  | *β* | *CI* | *β* | *CI* | *β* | *CI* | *β* | *CI* |
| Intercept | 0.00 | [-0.02, 0.02] | 0.00 | [-0.02, 0.02] | 0.00 | [-0.02, 0.02] | 0.00 | [-0.02, 0.02] |
| Fixed effects: Religiosity |  |  |  |  |  |  |  |  |
| Maternal religiosity | **-.03**** | [-.05, -.01] | **-.02*** | [-.04, .00] | **-.02*** | [-.02, .00] | **-.02*** | [-.04, .00] |
| Religiosity norm | - | - | **-.10***** | [-.13, -.06] | **.04**** | [.01, .07] | **-.03**** | [-.05, -.01] |
| Fixed effects: Context |  |  |  |  |  |  |  |  |
| Household income | **-.06***** | [-.08, -.03] | - | - | - | - | **-** | - |
| Welfare recipient | **.07***** | [.05, .09] | - | - | - | - | - | - |
| Unmet healthcare | **.03**** | [.01,.05] | - | - | **-** | - | - | - |
| Income norm | - | - | .03 | [.00, .07] | **-** | - | - | - |
| Welfare norm | - | - | - | - | **.14***** | [.11, .16] | - | - |
| Healthcare norm | - | - | - | - | **-** | - | **-.08***** | [-.10, -.06] |
| Random effects: Child |  |  |  |  |  |  |  |  |
| Residual (σ²) | 0.83 |  | .83 |  | .82 |  | .82 |  |
| Intercept (τ_00_) | 0.15 |  | 0.17 |  | 0.17 |  | 0.17 |  |
| *R²* _fixed effects_ | .01 |  | .01 |  | .01 |  | .01 |  |
| *R²* _total_ | .17 |  | .17 |  | .17 |  | .17 |  |

Note. Because norm variables are computed as means per study wave, regression models would run into multicollinearity issues when trying to fit various norm variables in a model. Therefore, only one context norm variable is included in each model. Coefficients for norm variables should be interpreted with caution. N _children_ = 3,544, N _Observations_ = 11,377; * p < .05; ** p < .01; *** p < .001.

Supplementary Table S7. Cross-lagged panel-model controlled for context factors

| Path (predictor → outcome) | *ꞵ* | *p* |
| --- | --- | --- |
| *a* paths |  |  |
| Anxiety at age 3 → Anxiety at age 15 | .11 | <.001*** |
| Religiosity at age 3 → Religiosity at age 15 | .41 | <.001*** |
| *c* paths |  |  |
| Anxiety at age 3 → Religiosity at age 15 | -.05 | .013* |
| Religiosity at age 3 → Anxiety at age 15 | -.01 | .576 |

Note. * p < .05; ** p < .01; *** p < .001.

**Supplementary Figures**

Supplementary Figure S1. Variable loadings on the principal components (PCs) per study wave.


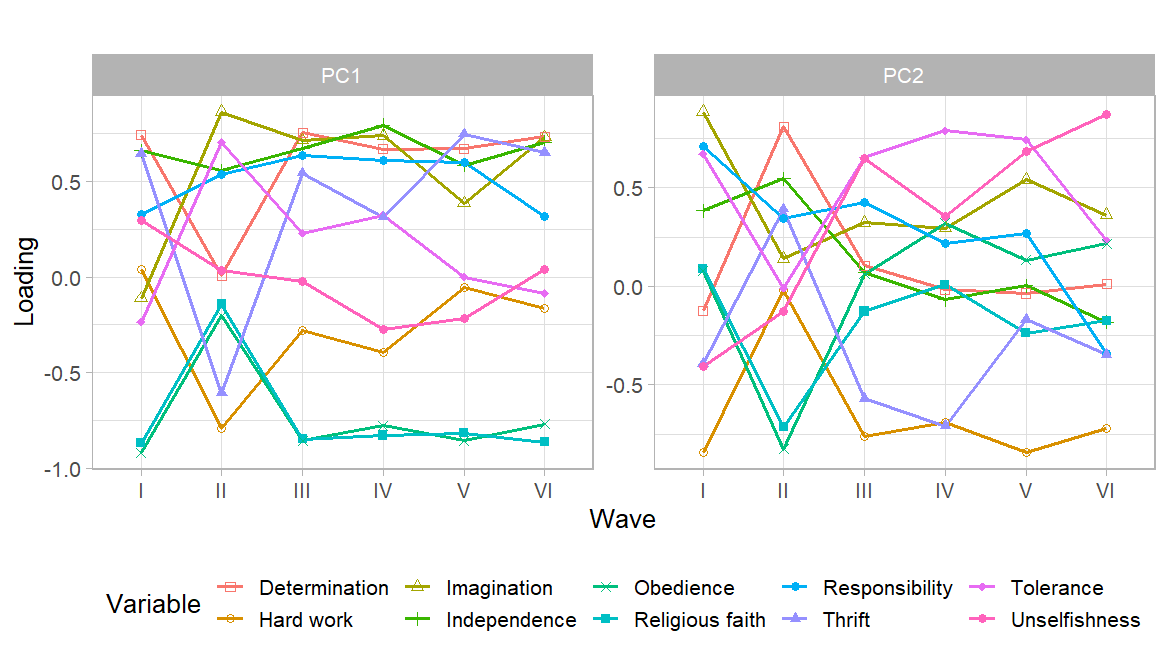


Supplementary Figure S2. Bivariate cross-temporal correlations for Western and non-Western countries.


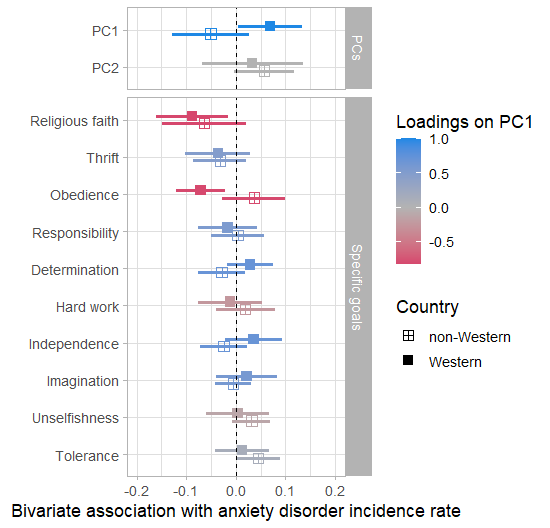


Supplementary Figure S3. Bivariate cross-temporal correlations for high-income and not high-income countries.


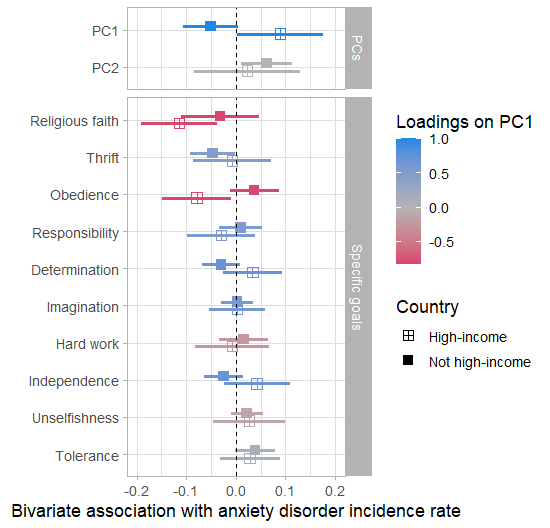


**Supplementary Discussion**

**Discussion of between-country results**

Highest anxiety incidence rates were observed in Norway, Iran, Switzerland, Cyprus, and Germany, whereas the lowest rates were found in Kazakhstan, India, Kyrgyzstan, Vietnam, and Pakistan. This distribution closely mirrors global epidemiological patterns. Meta-analytic evidence indicates that while estimated adult anxiety prevalence across world regions typically ranges between 5.3% and 8.0%, European and Anglophone countries show distinctly higher rates, around 10.4%. One explanation lies in the presence of well-developed mental health care systems in these countries, characterized by standardized diagnostic frameworks, routine screening, and widespread public awareness. Such infrastructures increase the likelihood that internalizing symptoms are recognized, classified, and recorded. By contrast, when Western diagnostic standards are applied in non-Western contexts, anxiety disorders may remain underdetected, contributing to lower reported prevalence and incidence (Baxter et al., 2013).

Iran stands out as an outlier in this high-incidence cluster. Unlike the other countries with elevated rates, Iran is not a high-income Western society, yet it shows comparably high anxiety incidence. A plausible explanation lies in macro-level stressors. In particular, international sanctions and prolonged economic instability have been linked to increased health care costs and reduced access to medical and psychological services, especially for low-income populations. These conditions are associated with unmet treatment needs, chronic stress exposure, and higher comorbidity, all of which may elevate population-level anxiety incidence (Mohamadi et al., 2024).

The lowest incidence rates cluster in Asian countries, specifically in Central Asia (Kazakhstan, Kyrgyzstan) and South and Southeast Asia (India, Pakistan, Vietnam). This geographic pattern aligns with previous work showing systematically lower prevalence estimates in many low- and middle-income regions. One contributing factor could be stigma: cross-national research indicates that stigmatizing attitudes toward mental illness are approximately twice as common in low- and middle-income countries compared to high-income countries. Elevated stigma can reduce help-seeking, discourage open reporting of psychological symptoms, and increase the tendency to frame distress in non-psychiatric terms. As a result, worry, fearfulness, or bodily complaints may be interpreted as normal reactions to life circumstances rather than as indicators of a mental disorder, lowering formal identification and survey-based reporting (Alonso et al., 2008).

The most independent socialization norms were found in Japan, Germany, Sweden, Norway, and South Korea. The most interdependent socialization norms were found in Ghana, Nigeria, Iraq, Egypt and Zimbabwe. Respectively, independence-associated socialization goals were most pronounced in countries like Norway and Slovenia while interdependence-associated socialization goals were most pronounced in Indonesia and Ghana.

This pattern closely follows established cultural models of child development. Northern and Western European societies have long been described as emphasizing autonomy, self-expression, and early self-regulation (Bornstein, 2012). In these contexts, a “competent child” is expected to act independently, articulate preferences, and manage emotions with limited adult intervention. The clustering of Japan and South Korea with these countries reflects a form of independence centered on early behavioral self-control and educational achievement rather than on individualism in a Western sense. The socialization goals “determination” and “responsibility” may therefore have been endorsed by East Asian parents primarily as qualities that support educational attainment (Jaramillo et al., 2017). Although East Asian societies are often described as interdependent, economic and educational transformations have strengthened the salience of such independence-related traits (Greenfield, 2009). At the same time, there is evidence that expectations for children’s self-discipline, perseverance, and autonomous academic engagement have been present in these contexts for several decades (Shek & Chan, 1999), indicating that these values are not merely recent adaptations but part of a longer-standing emphasis on educational success. Independent values (e.g., individual success of a child) may interact with interdependent values (e.g., filial piety), with both orientations coexisting in East Asian cultures and jointly shaping the broader meaning of socialization goals (e.g., interdependent family structures where children are encouraged to financially support their aging parents).

In contrast, countries in Sub-Saharan Africa and the Middle East often emphasize obedience and religious faith as central socialization goals. Comparative and ethnographic research describes these contexts as prioritizing respect for parental authority, moral conduct, and adherence to norms, with competence defined in terms of fulfilling social and moral obligations rather than asserting individual preferences (Keller, 2018). The prominence of these goals in countries such as Ghana, Nigeria, Iraq, Egypt, and Zimbabwe thus reflects a model of development in which children are socialized to become reliable members of relational and faith-based communities.

Data access: Both raw data sources are publicly available and can be accessed freely online at the following websites.

- <https://www.worldvaluessurvey.org/WVSOnline.jsp>
- <https://vizhub.healthdata.org/gbd-results/>

**Supplementary discussion references**

Alonso, J., Buron, A., Bruffaerts, R., He, Y., Posada‐Villa, J., Lepine, J., Angermeyer, M. C., Levinson, D., de Girolamo, G., Tachimori, H., Mneimneh, Z. N., Medina‐Mora, M. E., Ormel, J., Scott, K. M., Gureje, O., Haro, J. M., Gluzman, S., Lee, S., Vilagut, G., … von Korff, M. (2008). Association of perceived stigma and mood and anxiety disorders: results from the World Mental Health Surveys. Acta Psychiatrica Scandinavica, 118(4), 305–314. https://doi.org/10.1111/j.1600-0447.2008.01241.x

Baxter, A. J., Scott, K. M., Vos, T., & Whiteford, H. A. (2013). Global prevalence of anxiety disorders: a systematic review and meta-regression. Psychological Medicine, 43(5), 897–910. https://doi.org/10.1017/S003329171200147X

Bornstein, M. H. (2012). Cultural Approaches to Parenting. Parenting, 12(2–3), 212–221. https://doi.org/10.1080/15295192.2012.683359

Greenfield, P. M. (2009). Linking Social Change and Developmental Change: Shifting Pathways of Human Development. Developmental Psychology, 45(2), 401–418. https://doi.org/10.1037/A0014726

Jaramillo, J. M., Rendón, M. I., Muñoz, L., Weis, M., & Trommsdorff, G. (2017). Children’s Self-Regulation in Cultural Contexts: The Role of Parental Socialization Theories, Goals, and Practices. Frontiers in Psychology, 8. https://doi.org/10.3389/fpsyg.2017.00923

Keller, H. (2018). Universality claim of attachment theory: Children’s socioemotional development across cultures. Proceedings of the National Academy of Sciences, 115(45), 11414–11419. https://doi.org/10.1073/pnas.1720325115

Mohamadi, E., Kraemer, A., Majdzadeh, R., Mohamadzade, M., Mohammadshahi, M., Kiani, M. M., Ebrahimi, F., Mostafavi, H., Olyaeemanesh, A., & Takian, A. (2024). Impacts of economic sanctions on population health and health system: a study at national and sub-national levels from 2000 to 2020 in Iran. Globalization and Health, 20(1), 81. https://doi.org/10.1186/s12992-024-01084-2

Shek, D. T. L., & Chan, L. K. (1999). Hong Kong Chinese Parents’ Perceptions of the Ideal Child. The Journal of Psychology, 133(3), 291–302. https://doi.org/10.1080/00223989909599742
